# High microbiota reactivity of adult human intestinal IgA requires somatic mutations

**DOI:** 10.1084/jem.20200275

**Published:** 2020-07-08

**Authors:** Johanna Kabbert, Julia Benckert, Tim Rollenske, Thomas C.A. Hitch, Thomas Clavel, Vuk Cerovic, Hedda Wardemann, Oliver Pabst

**Affiliations:** 1 Institute of Molecular Medicine, Rheinisch-Westfälische Technische Hochschule Aachen University, Aachen, Germany; 2 Max Planck Research Group Molecular Immunology, Max Planck Institute for Infection Biology, Berlin, Germany; 3 B Cell Immunology, German Cancer Research Centre, Heidelberg, Germany; 4 Functional Microbiome Research Group, Institute of Medical Microbiology, Rheinisch-Westfälische Technische Hochschule Aachen University, Aachen, Germany; 5 Charité–Universitätsmedizin Berlin, corporate member of Freie Universität Berlin, Humboldt-Universität zu Berlin, and Berlin Institute of Health, Berlin, Germany

## Abstract

The gut is home to the body’s largest population of plasma cells. In healthy individuals, IgA is the dominating isotype, whereas patients with inflammatory bowel disease also produce high concentrations of IgG. In the gut lumen, secretory IgA binds pathogens and toxins but also the microbiota. However, the antigen specificity of IgA and IgG for the microbiota and underlying mechanisms of antibody binding to bacteria are largely unknown. Here we show that microbiota binding is a defining property of human intestinal antibodies in both healthy and inflamed gut. Some bacterial taxa were commonly targeted by different monoclonal antibodies, whereas others selectively bound single antibodies. Interestingly, individual human monoclonal antibodies from both healthy and inflamed intestines bound phylogenetically unrelated bacterial species. This microbiota cross-species reactivity did not correlate with antibody polyreactivity but was crucially dependent on the accumulation of somatic mutations. Therefore, our data suggest that a system of affinity-matured, microbiota cross-species–reactive IgA is a common aspect of SIgA–microbiota interactions in the gut.

## Teaser

The authors reveal a mechanism of SIgA–microbiota interaction in the adult human gut. They describe antibodies with a high capacity to bind phylogenetically distant members of the microbiota. Such cross-species reactivity does not correlate with antibody polyreactivity but crucially depends on somatic mutations.

## Introduction

IgA-secreting plasma cells constitute a major leukocyte population in the human gut. IgM-secreting plasma cells are also present (Magri et al., 2017) and, in patients with inflammatory bowel disease (IBD), high concentrations of IgG are detectable in the intestinal lumen (Macpherson et al., 1996). However, the specificity of intestinal antibodies for luminal antigen, including components of the gut microbiota, are largely unknown (Pabst and Slack, 2020).

Polymeric IgA and IgM are actively transported across the epithelial cell layer into the gut lumen by the polymeric Ig receptor. During this process, a fragment of the polymeric Ig receptor becomes covalently bound to the antibody to generate a hybrid molecule referred to as secretory Ig (SIg; Brandtzaeg, 2009; Stadtmueller et al., 2016). In the human gut lumen, different antibody isotypes show differential binding to members of the microbiota, resulting in selective coating by SIgA, SIgM, and IgG (Fadlallah et al., 2019; Magri et al., 2017; Palm et al., 2014). Ig coating of a given bacterial species can result in different outcomes ranging from extinction/loss of the Ig-coated bacteria (typically observed only for pathogens), to no detectable effects on colonization levels, or even an increase in colonization (Donaldson et al., 2018; Moor et al., 2017; Peterson et al., 2015). The fraction of SIgA-coated members of the gut microbiota varies along the intestinal tract, ranging from ∼60% of total bacteria in the proximal small intestine to ∼10% in feces (Bunker et al., 2015; van der Waaij et al., 1996). Notably, many taxa coated by SIgA in the colon are also coated in the small intestine, suggesting that microbiota-reactive IgA is primarily induced in the proximal gut segments (Bunker et al., 2015).

Recently, the functional properties of intestinal bacteria coated by endogenous polyclonal IgA have been reported. Sort purification of SIgA-coated bacteria followed by 16S ribosomal RNA (rRNA) gene amplicon sequencing revealed enrichment of distinct bacterial taxa in IgA-coated fractions (Bunker et al., 2015; Kau et al., 2015; Palm et al., 2014). Mice colonized with SIgA-coated gut bacteria showed increased susceptibility to colitis (Palm et al., 2014) and enhanced diet-dependent enteropathy (Kau et al., 2015) compared with animals colonized with noncoated bacteria. These observations show that SIgA coating is linked to distinct functional properties of gut bacteria.

However, we are largely ignorant when it comes to describing defined antigens bound by IgA or the precise mechanisms of how IgA binds to members of the microbiota. SIgA can bind to microorganisms by canonical Fab-dependent binding as well as noncanonical interactions between antibody and microbiota (Pabst and Slack, 2020). Noncanonical interactions are unaffected by somatic mutations in the Fab region of the antibody and rely on glycan moieties associated with the Fc part of the antibody and the secretory component (Mathias and Corthesy, 2011; Pabst and Slack, 2020). The relevance of such noncanonical binding to members of the microbiota is not well defined.

To investigate the specificity of IgA for the microbiota, a recent study in mice showed that single mAbs bound to diverse but defined subsets of microbial populations (Bunker et al., 2017). This binding profile was associated with antibody polyreactivity and was independent of somatic mutations (Bunker et al., 2017). Yet, in the human gut, germline (GL)-encoded IgA is virtually absent, and IgA-secreting plasma cells are characterized by high numbers of somatic mutations (Barone et al., 2011; Benckert et al., 2011; Lindner et al., 2015). Indeed, human mAbs directed against LPS O-antigens of *Klebsiella pneumoniae* recognize intestinal microbes, and this binding required somatic mutations (Rollenske et al., 2018). We suggest referring to this phenomenon of single mAbs binding to different bacterial species as cross-species reactivity (Pabst and Slack, 2020). Mechanistically, cross-species reactivity is not fully understood.

Here, we characterized a panel of IgG and IgA mAbs derived from human small intestine of both healthy donors (HDs) and Crohn’s disease (CD) patients. This approach allowed us to contrast the microbiota-binding capacity of IgA and IgG, but also to compare antibody specificity for members of the gut microbiota under healthy and inflamed conditions. Our data demonstrate that microbiota binding is a frequent property of IgA and IgG under both healthy and inflamed conditions. In most cases, the binding capacity and spectrum of human IgA was crucially dependent on somatic mutations and did not correlate with polyreactivity. We therefore suggest that, in adult humans, a system of affinity matured, microbiota cross-species–reactive IgA is a common aspect of SIgA–microbiota interactions in the gut.

## Results

### Microbiota-reactive IgA and IgG antibodies are abundant in the adult human small intestine

To investigate the microbiota reactivity and specificity of Fab-dependent canonical binding of human intestinal antibodies under steady-state and inflammatory conditions, we generated two collections of recombinant mAbs. A first set of 162 antibodies was derived from the terminal ileum of three HDs, reported previously (Benckert et al., 2011). A second collection of 118 antibodies was obtained and generated from patients with IBD (CD; Table S1). For both HD and CD donors, IgA- and IgG-derived antibodies were cloned from single lamina propria IgA^+^CD38^+^CD27^+^ and IgG^+^CD38^+^CD27^+^ plasmablasts/plasma cells, respectively. To allow for unbiased comparison of gut microbiota binding of IgA- and IgG-derived antibodies, all mAbs were expressed as human IgG1 in mammalian cells (Benckert et al., 2011; Tiller et al., 2008). Thus, differences between individual antibodies with respect to microbiota-binding capacity and specificity reflect differences in canonical Fab-dependent antigen binding, whereas potential noncanonical binding properties of endogenous SIgA are not captured in this approach. In total, we generated and screened 280 human mAbs for their microbiota reactivity (IgA, 105 from HD and 84 from CD donors; IgG, 57 from HD and 34 from CD donors).

**Figure 1.**
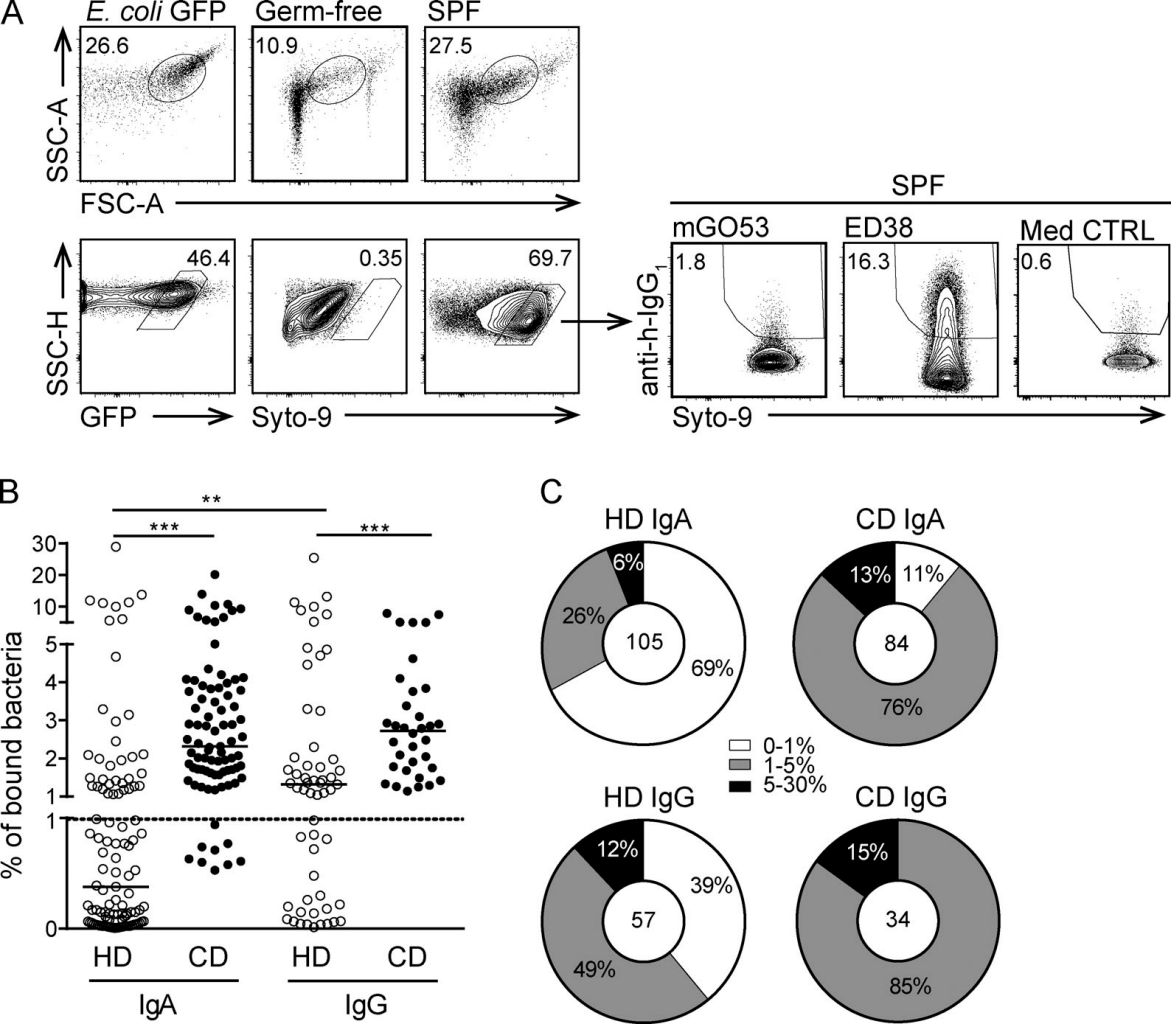
**Microbiota-reactive IgA antibodies are abundant in the adult human small intestine. (A)** IgA-free microbes were isolated from feces of SPF Rag2-deficient mice. Gates were set according to in vitro–cultured GFP-expressing *E. coli* and fecal material isolated from germ-free mice (top row). GFP signal or Syto9 nucleic acid dye were used to identify bacteria (bottom row). Anti-human IgG1 antibody was used to detect mAb-positive fecal bacteria. Representative staining is depicted for previously described low (mGO53) and high (ED38) polyreactive control antibodies (Meffre et al., 2004; Wardemann et al., 2003) and staining with supernatant from nontransfected HEK-cells (Med CTRL) on feces from SPF Rag2-deficient mice. **(B and C)** Two collections of antibodies derived from IgA- or IgG-expressing plasmablasts/plasma cells from HDs (Benckert et al., 2011) or CD patients were screened for reactivity to microbes isolated from SPF Rag2-deficient mice. **(B)** Symbols represent individual antibodies (162 HD mAbs and 118 CD mAbs) assayed in one representative experiment. Binding to <1% of bacteria was considered background (dashed line). Groups were compared by Kruskal–Wallis and post hoc Mann–Whitney *U* test (**, P < 0.001; ***, P < 0.0001). **(C)** Antibodies were categorized according to the following binding capacities: no/low (0–1% of bacteria), intermediate (1–5%), and high (≥5%). FSC-A, forward scatter area; SSC-A, side scatter area; SSC-H, side scatter height.

To avoid potential competition for epitope binding between these recombinant antibodies and endogenous IgA, fecal samples from Rag2-deficient mice, which lack B cells and therefore soluble Ig, were used. Microbial cells were identified by flow cytometry based on their scatter profiles and staining intensity with the DNA-binding dye Syto9. Material isolated from feces of germ-free mice and in vitro–cultured *Escherichia coli* was used to establish precise gating of bacteria (Fig. 1 A).

Bacterial staining with the control antibody (mG053; Wardemann et al., 2003) and antibody-free cell culture supernatant showed variable background staining (0.52 ± 0.31, mean ± SD, *n* = 36). Thus, to exclude the possibility of false positives, we considered binding of ≥1% total microbial cells as the threshold of antibody reactivity. This definition of antibody-positive populations does not exclude the possibility of true binding of antibodies with lower binding capacity to bacteria.

Both IgA and IgG mAbs showed a wide spectrum of microbiota reactivity, including antibodies with no microbiota binding and antibodies binding >5% of all fecal bacteria (Fig. 1, B and C). We therefore categorized antibodies to have no/low (0–1% of bacteria), intermediate (1–5%), and high (>5%) binding capacity for gut bacteria. Antibodies with high binding capacity for gut bacteria were present among mAbs of both IgA and IgG origin (Fig. 1 B). Notably, high microbiota-binding capacity was observed in both collections of mAbs but clearly more pronounced in antibodies derived from CD patients compared with HD-derived antibodies (Fig. 1 C). We therefore conclude that microbiota-binding capacity is a characterizing property of a substantial proportion of human intestinal IgA and IgG antibodies in both healthy and inflamed gut.

### Intestinal IgA antibodies bind diverse but distinct groups of gut bacteria

**Figure S1.**
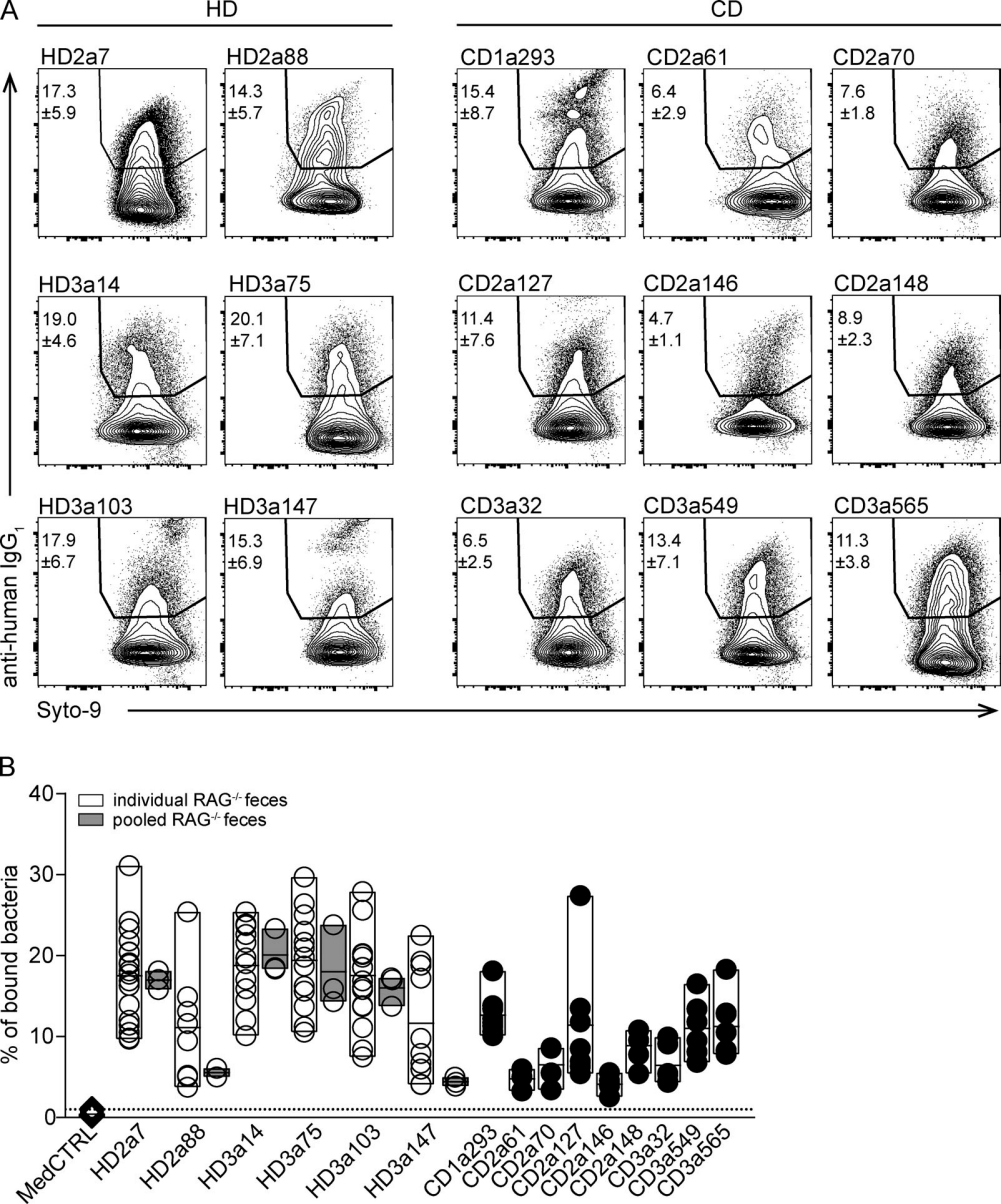
**High-microbiota-reactive IgA antibodies bind a major fraction of the intestinal microbiota. (A)** Representative FACS plots demonstrating staining of bacteria (gated on forward/side scatter profile and Syto9-positive events) with selected high-microbiota-reactive HD and CD IgA-derived mAbs. Numbers indicate mean percentage of gated events ± SD of at least four independent experiments. **(B)** Binding capacity of selected HD (open circles) and CD (black circles) mAbs to microbiota over a large set of independent experiments. For HD mAbs only, binding to bacteria is displayed for unrelated fecal material (white bars) or for aliquots of pooled identical fecal material (gray bars) of three independent experiments. Single dots represent independent experiments; bars represent minimum to maximum values of microbiota reactivity for individual mAbs.

To further characterize the binding specificity and mechanisms regulating the binding of human intestinal antibodies to microbiota, we selected 15 high-microbiota-reactive mAbs cloned from IgA plasmablasts/plasma cells (six from HDs, nine from CD patients; Fig. 1 C) for further analyses (Fig. S1 A). Two of the selected HD IgA mAbs are clonally related (HD3a14 and HD3a75), suggesting that the two antibodies might show similar properties and specificity.

All selected antibodies showed high microbiota-binding capacity across independent experiments performed with murine donor feces obtained from different cages and collected at different times (Fig. S1 B). Notably, across the experiments, we observed marked variability in the percentage of antibody-bound bacteria. Performing experiments on aliquots of pooled fecal material reduced variability (Fig. S1 B), suggesting that microbial variations in the fecal material contributed to the observed variability in antibody binding.

**Figure 2.**
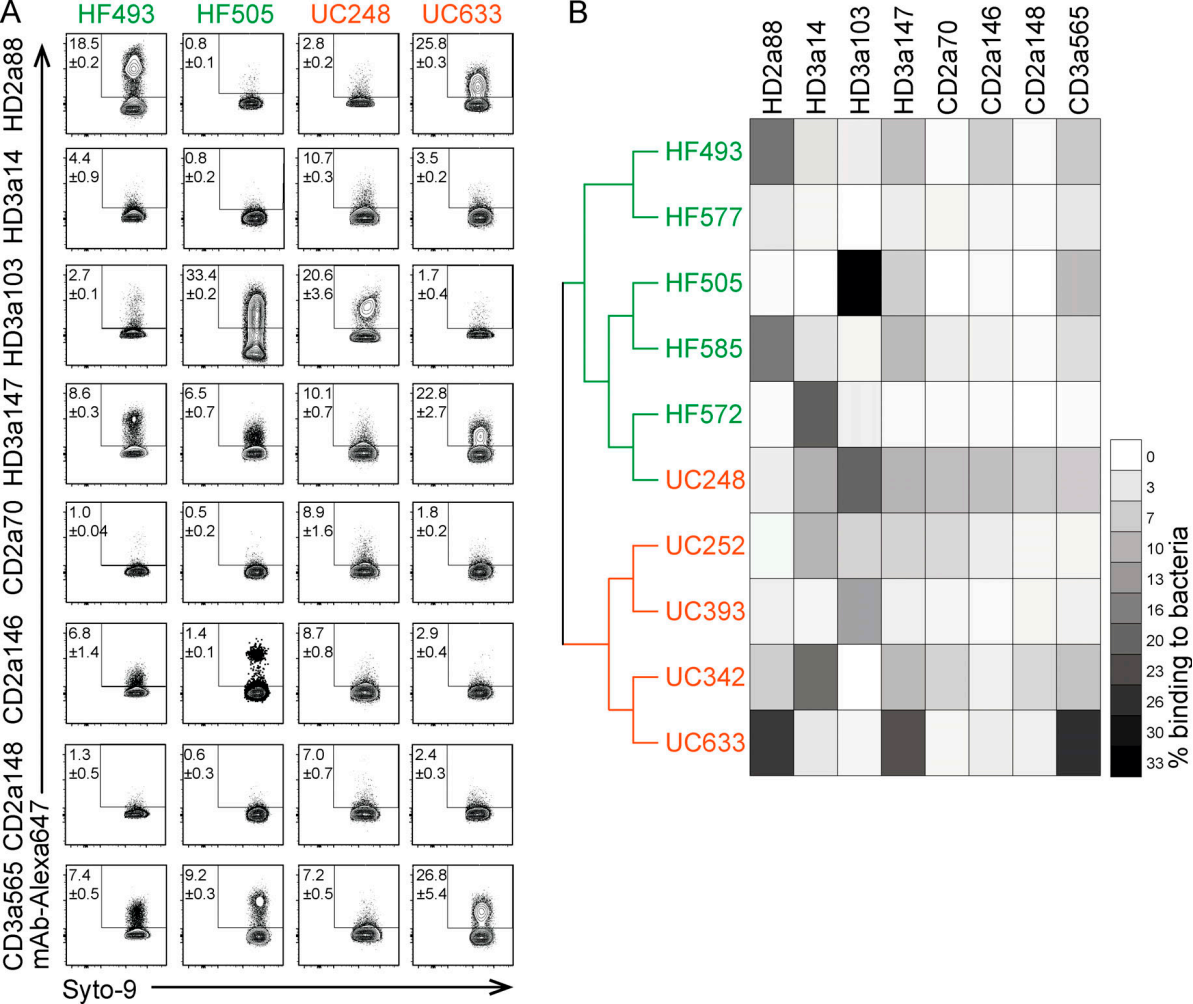
**Binding capacity of intestinal IgA antibodies to human gut bacteria shows donor-dependent variability.** Bacteria were isolated from fecal samples of five healthy individuals (HF) and five ulcerative colitis (UC) patients and stained with directly conjugated mAbs. **(A)** Representative FACS plots of microbiota reactivity of mAbs to human gut bacteria. Microbes were stained with Syto9 nucleic acid dye, and microbiota reactivity of AF647 directly conjugated mAbs was assessed by flow cytometry. Data are representative of two independent experiments (mean ± SD). **(B)** Fecal samples were characterized by 16S rRNA amplicon sequence analysis. Dendrogram clustering of individual fecal samples is based on their bacterial genomic sequence composition. Heat map depicts binding capacity of single mAbs to bacteria isolated from respective HF and UC donors (organized as a dendrograms) displayed as mean of two independent experiments.

**Figure 3.**
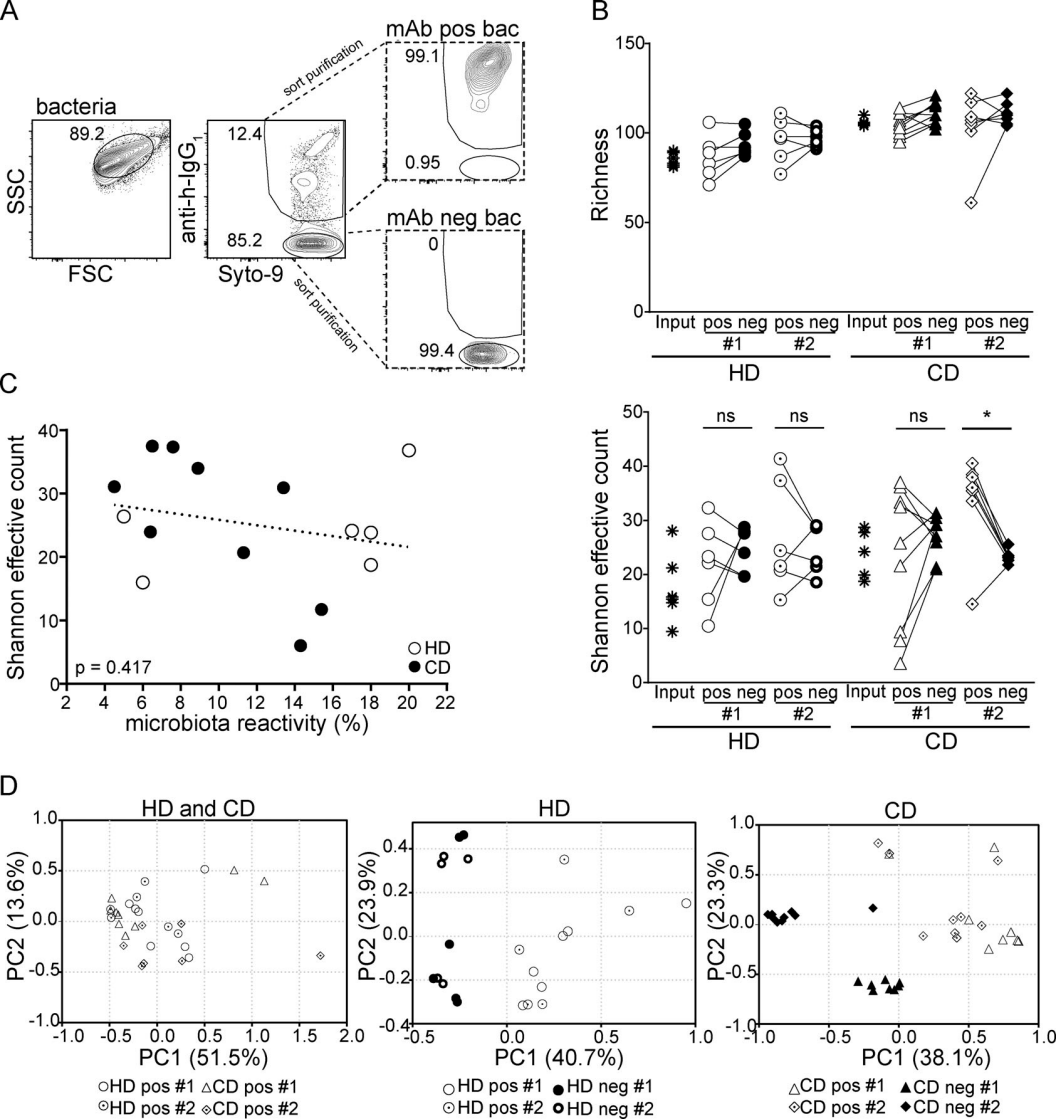
**Intestinal IgA antibodies bind diverse groups of gut bacteria. (A)** Representative illustration of antibody-positive (mAb pos bac) and antibody-negative bacteria (mAb neg bac) obtained by cell sorting. The diversity and composition of sorted bacteria were characterized by 16S rRNA amplicon sequencing. **(B)** α-Diversity parameters (richness and Shannon effective count) for sorted antibody-positive (pos) and -negative (neg) bacteria. Two separate experiments, #1 and #2, were performed. Groups were compared by one-way ANOVA and paired Wilcoxon test (*, P < 0.05; ns, not significant). **(C)** Correlation analysis considering the Shannon effective count as displayed in B and bacteria binding in percent. Symbols represent the mean of two independent experiments. **(D)** β-Diversity displayed as PCoA of generalized Unifrac distances. The plots depict comparison between antibody-positive samples from HDs and CD patients (left plot) and between antibody-positive and -negative samples from HDs (middle) and CD patients (right). FSC, forward scatter; SSC, side scatter.

Major variability with respect to microbiota-binding profiles was also apparent when testing high-microbiota-reactive mAbs for binding to bacteria present in human feces. Screening of human fecal samples required direct fluorochrome conjugation of selected mAbs because of the high background binding of the anti-human IgG1 secondary antibody to human fecal bacteria (likely reflecting endogenous IgG coating of human gut bacteria). To cover a range of different microbiota configurations, mAbs were tested against five samples obtained from HDs and five samples from IBD patients. Indeed, 16S rRNA sequencing revealed notable differences in microbiota β-diversity between healthy and IBD samples (not depicted). Seven of eight antibodies selected for high microbiota reactivity based on the screening of Rag2-deficient mouse feces also showed high reactivity to bacteria isolated from several human fecal samples (Fig. 2, A and B). However, the binding profile of mAbs differed greatly between individual samples (Fig. 2 B). Such distinct binding profiles likely reflect differences in microbial species composition but may also be influenced by additional factors modulating antibody binding capacity, such as transcriptomic state of gut microbiota and environmental modification of antibody epitopes. Irrespectively, these data show that high microbiota reactivity is a feature shared across a broad range of fecal samples. Considering the marked variability between different human donors with respect to the percentage of mAb binding to bacteria as well as the uncertainty with respect to the underlying factors affecting mAb binding and the impact of endogenous Ig coating the microbiota, we chose to further characterize mAb binding to mouse microbiota. Aiming at a robust description of antibody-bound taxa (Fig. S1 B), two separate sets of experiments were performed using feces from non-cohoused mice. To investigate which microbial taxa were targeted by the selected mAbs, bacteria bound and unbound by high-microbiota-reactive mAbs were sort-purified by FACS and subjected to 16S rRNA gene amplicon sequencing (Fig. 3 A).

We first examined α-diversity parameters (richness and Shannon effective counts) to assess the complexity of input and sorted bacterial populations (Fig. 3 B). No major differences were observed for richness, implying that each individual mAb bound multiple bacterial taxa. Antibody-positive samples showed a notable spread in Shannon effective counts (which integrates the evenness of molecular species and can be considered as a proxy for numbers of dominant species), suggesting a potential heterogeneity of antibody binding profiles. Notably, α-diversity of antibody-positive samples did not correlate with bacteria binding capacity (Fig. 3 C), i.e., samples sorted with antibodies binding to a comparably smaller percentage of all gut bacteria showed a similarly high diversity as samples purified with antibodies binding to a very large proportion of all gut bacteria. This further underlines that antibodies with high binding capacities to gut microbiota show distinct binding specificities.

To assess and visualize differences between samples (β-diversity), we used principal coordinates analysis (PCoA) based on generalized UniFrac distances (Chen et al., 2012). Comparison of antibody-positive samples of HD- and CD donor–derived antibodies, i.e., healthy and inflamed intestine, revealed largely overlapping binding profiles to gut bacterial populations (Fig. 3 D, left panel). This indicates that, although CD is often accompanied by major shifts in the microbiota composition (Fadlallah et al., 2018), gut inflammation is not necessarily associated with an extensive shift in IgA specificity for particular members of the microbiota. In contrast, β-diversity analysis revealed major differences between antibody-positive and antibody-negative bacterial fractions across both sets of experiments (Fig. 3 D, middle and right panel). In conclusion, both α- and β-diversity analyses indicate that single monoclonal intestinal antibodies do not enrich for individual bacterial species but instead specifically bind broadly to distinct groups of gut bacteria.

### Intestinal IgA shows cross-species reactivity

**Figure S2.**
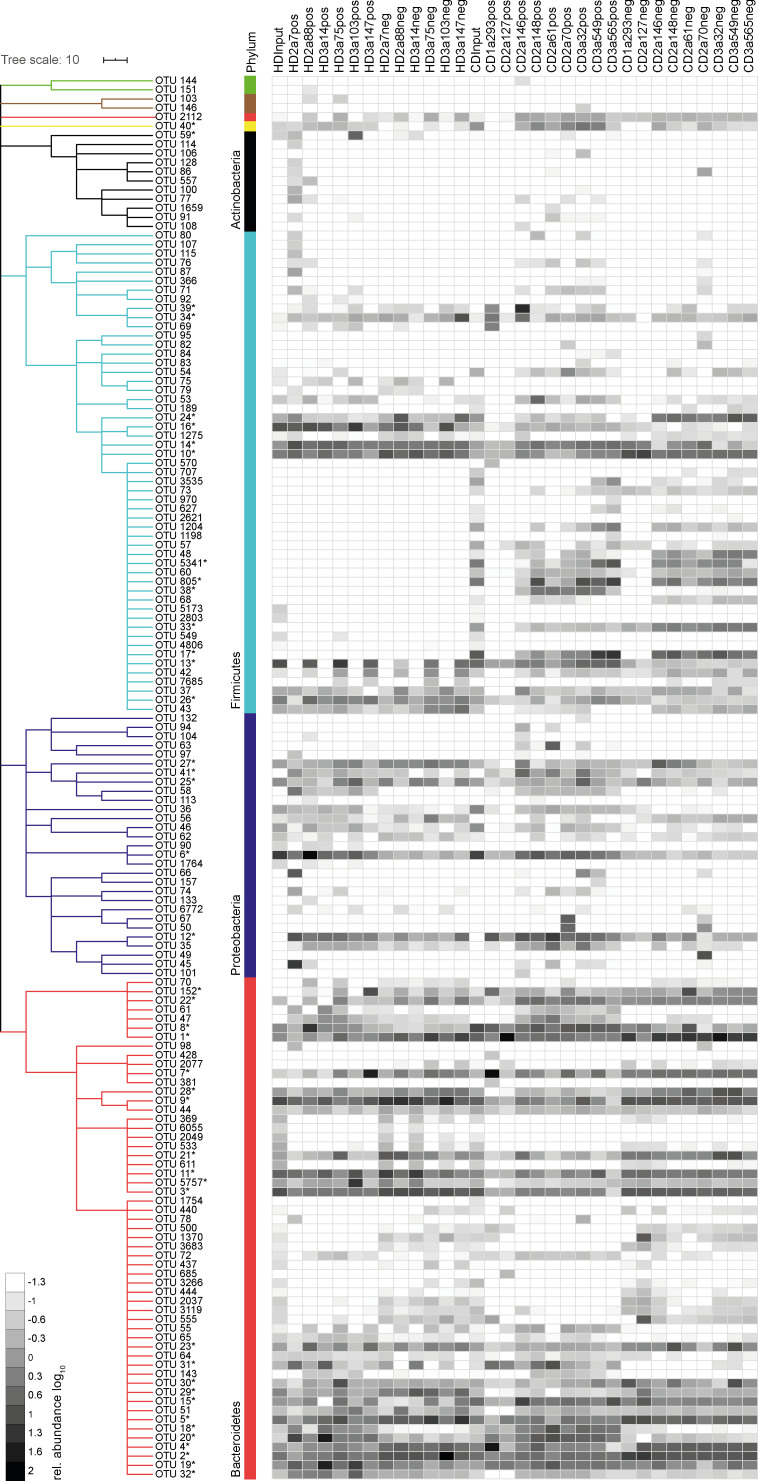
**Intestinal IgA binds to high- and low-abundance members of the microbiota.** Single OTUs are organized as a dendrogram (phylum classification in colors). Dendrogram clustering of OTUs (phylum to family) was based on the RDP taxonomic classification (Wang et al., 2007). OTUs marked by asterisks denote commonly or selectively targeted OTUs and are listed in Table S2. Taxonomic species annotation of asterisks marked OTUs was based on EZbiocloud (Yoon et al., 2017). Only OTUs occurring at a relative abundance ≥0.5% in any positive or negative fraction are displayed. Heat map shows log_10_-transformed relative abundances of OTUs.

**Figure 4.**
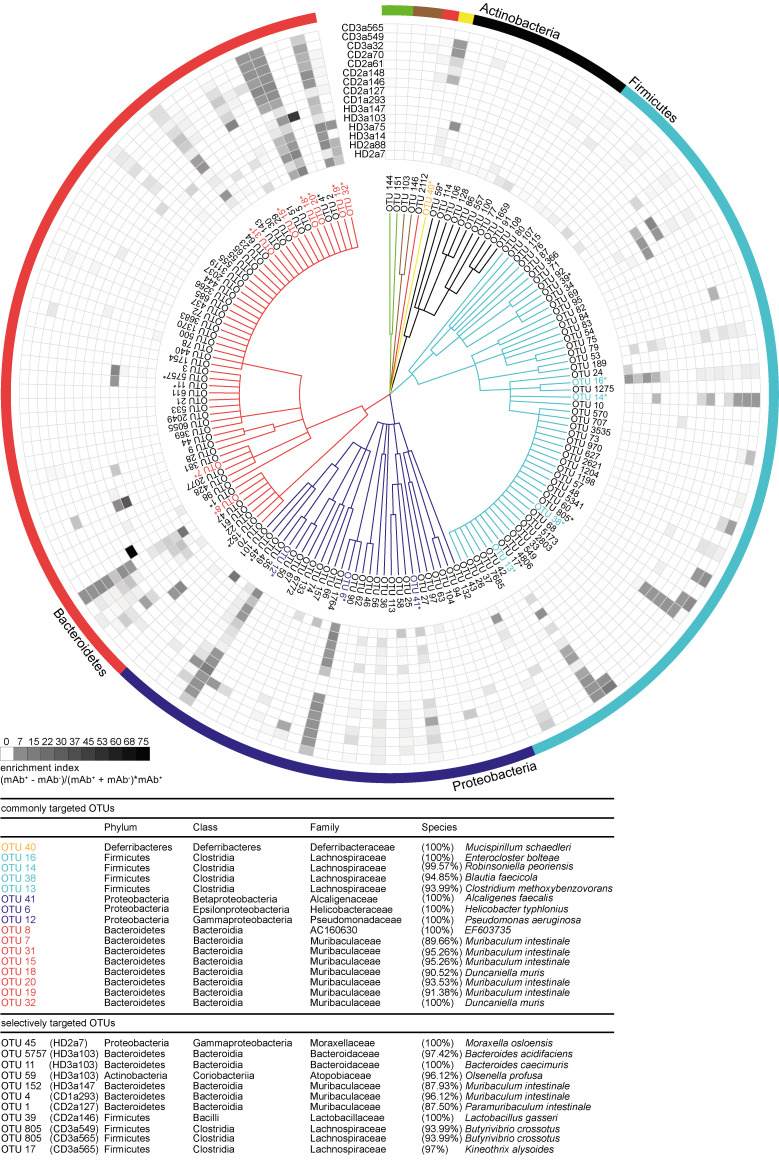
**High-microbiota-reactive intestinal IgA is cross-species reactive. **Single OTUs are organized as a dendrogram (phylum to family, annotations displayed in colors) based on the RDP taxonomic classification (Wang et al., 2007). Only OTUs occurring at a relative abundance of ≥0.5% in any positive fraction are displayed. Antibody specificity to a given OTU is displayed as enrichment index (as defined in the graph and determined as mean of two or more replicate experiments). 16 commonly targeted OTUs enriched twofold by ≥3 mAbs are indicated in colors, marked by asterisks, and listed below the figure. Selectively targeted OTUs (OTUs enriched by only a single mAb among the high-microbiota-reactive antibodies) are listed in black and marked by asterisks. The lineage and taxonomic identity (closest species with a valid name and corresponding 16S rRNA gene sequence identity) of these denoted OTUs were obtained using EZbiocloud (Yoon et al., 2017). Nonlisted phyla are as follows: Deinococcus-Thermus (green), Cyanobacteria (brown), and Deferribacteres (yellow). Taxonomic classifications of OTUs are listed in Table S2.

To identify bacterial taxa that explain the observed difference in β-diversity, we examined the relative abundance of individual operational taxonomic units (OTUs) in antibody-positive and -negative samples. For all mAbs investigated, antibody-positive samples contained multiple, phylogenetically distant, OTUs (Fig. S2). However, OTUs highly abundant in the antibody-positive samples were typically still detectable in the antibody-negative samples (Fig. S2). Therefore, we expressed the capacity of individual antibodies to enrich for distinct OTUs as an enrichment index. The index considers the ratio of relative OTU abundances in the antibody-positive and -negative samples plus the OTU abundance in the positive sample (see Materials and methods). Indeed, for all antibodies tested, the enrichment index highlighted several OTUs (Fig. 4). 16 OTUs were targeted by multiple antibodies, as defined by a twofold enrichment in the antibody-positive fraction for at least three individual mAbs tested, indicating that these OTUs are preferential targets of intestinal antibodies (Fig. 4 and Fig. S2). We will refer to these OTUs as commonly targeted OTUs.

Taxonomic assignment of these commonly targeted OTUs included microbial members of the main abundant phyla in the mouse gut, Firmicutes, Proteobacteria, Bacteroidetes, and Deferribacteres (Fig. 4 and Fig. S2) such as *Enterocloster bolteae* (OTU 16), *Mucispirillum schaedleri* (OTU 40), *Alcaligenes faecalis* (OTU 41), *Helicobacter typhlonius* (OTU 6), and several species that were identity matched to members of the family *Muribaculaceae* (OTU 7, 15, 18, 19, 20, 31, and 32). These taxa have previously been reported to be coated by endogenous IgA (Fadlallah et al., 2018; Magri et al., 2017; Palm et al., 2014; Sterlin et al., 2020). In contrast, some OTUs showed particularly strong enrichment by single antibodies. This pattern was observed for antibodies derived from HDs as well as CD patients (Fig. 4 and Fig. S2). Notably, OTUs with an overall low relative abundance were targeted by specific mAbs, e.g., *Moraxella osloensis* (OTU 45; HD2a7), *Bacteroides acidifaciens* (OTU 5757; HD3a103), *Muribaculum* sp. (OTU 4; CD1a293), and *Lactobacillus gasseri* (OTU 39; CD2a146). Therefore, IgA responses are not only directed against dominant members of the microbiota but also target underrepresented populations of the overall gut microbial communities. Our data demonstrate that individual intestinal antibodies can bind a broad range of microbial species while exhibiting unique binding profiles. Importantly, we observed phylogenetically unrelated taxa among selectively and commonly targeted OTUs, suggesting that single monoclonal human IgA antibodies can bind to unrelated groups of gut bacteria. We therefore conclude that cross-species reactivity is one characterizing property of human intestinal IgA in healthy and inflamed gut.

**Figure S3.**
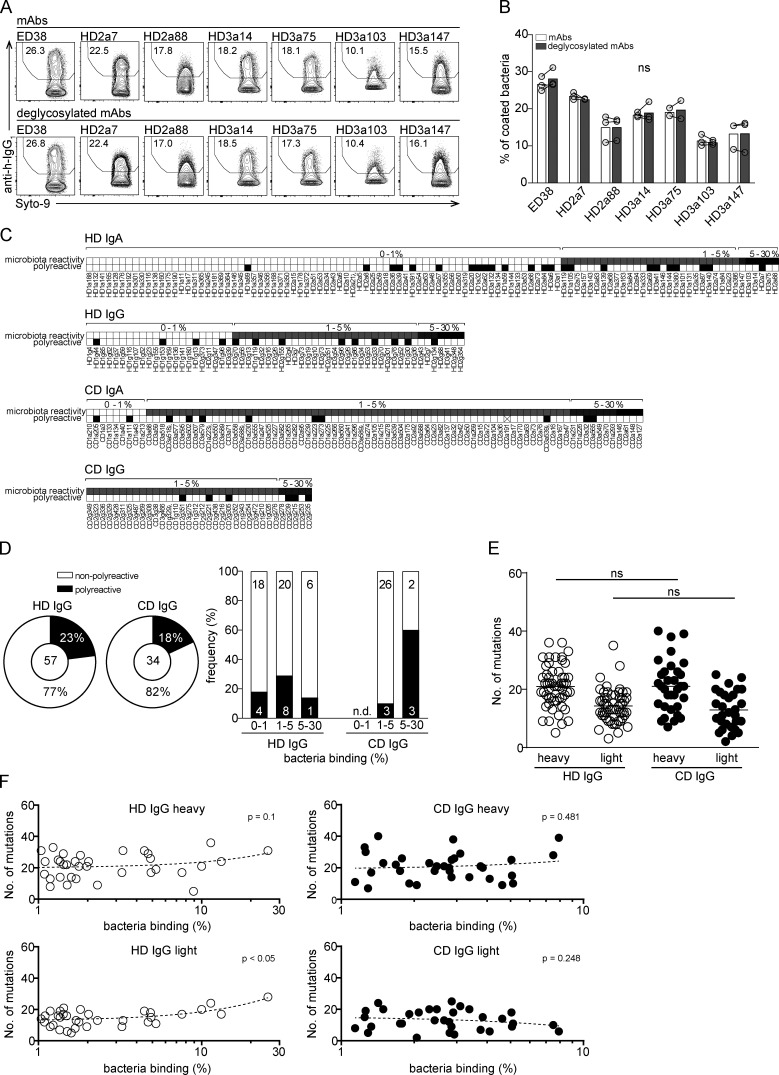
**High microbiota reactivity does not rely on glycosylation or polyreactivity but is characterized by high numbers of somatic mutations.** **(A)** Representative flow cytometry staining of bacteria (gated on forward/side scatter profile and Syto9-positive events) with selected unmodified or deglycosylated high-microbiota-reactive antibodies. Numbers indicate percentage of gated events. **(B)** Comparison of microbiota-binding capacity of glycosylated (white bars) and deglycosylated (gray bars) antibodies. Symbols denote independent experiments, bars show means, and lines connect data points obtained in the respective experiments. Significance was tested by paired Mann–Whitney *U* test; ns, not significant. **(C)** IgA and IgG antibodies were ranked according to their microbiota-binding capacity from non/low to intermediate and high reactivity (percentage binding depicted above plots). Antibodies were tested for binding to a set of seven nonrelated antigens and classified as polyreactive (black) and nonpolyreactive (white). **(D)** Antibodies were tested for binding to a set of nonrelated antigens and classified as polyreactive (black) and nonpolyreactive (white). Bar charts show the relative distribution of polyreactive and nonpolyreactive HD and CD IgG antibodies among antibodies with no/low, intermediate, and high microbiota reactivity. Correlation between polyreactivity and microbiota reactivity was determined by linear regression (HD IgG, P = 0.75; CD IgG, P = 0.02). **(E)** Numbers of somatic mutations in heavy and light chain V genes displayed for all IgG antibodies obtained from HDs and CD patients. **(F)** Microbiota-binding capacity displayed as percentage of bound microbiota for a given antibody was correlated to the number of somatic mutations in heavy and light chain V genes for all IgG antibodies from HDs and CD patients. P values were obtained by linear regression analysis.

The mAbs tested here were expressed as human IgG1 antibodies independently of their original isotype and subclass. Therefore, it seems unlikely that the binding patterns described herein rely on Fc-mediated interactions and glycan modifications. Nonetheless, glycan-mediated interactions between mAbs and individual members of the gut microbiota have been reported (Nakajima et al., 2018), and different mAbs may differ in their precise glycan modification. To directly determine the contribution of antibody glycan structures to microbiota binding, we deglycosylated a set of purified mAbs. Deglycosylation did not affect the overall binding capacity of mAbs (Fig. S3, A and B), suggesting that glycan modifications do not contribute to the observed microbiota-binding patterns.

### Somatic mutations but not polyreactivity confer cross-species binding and high microbiota reactivity

**Figure 5.**
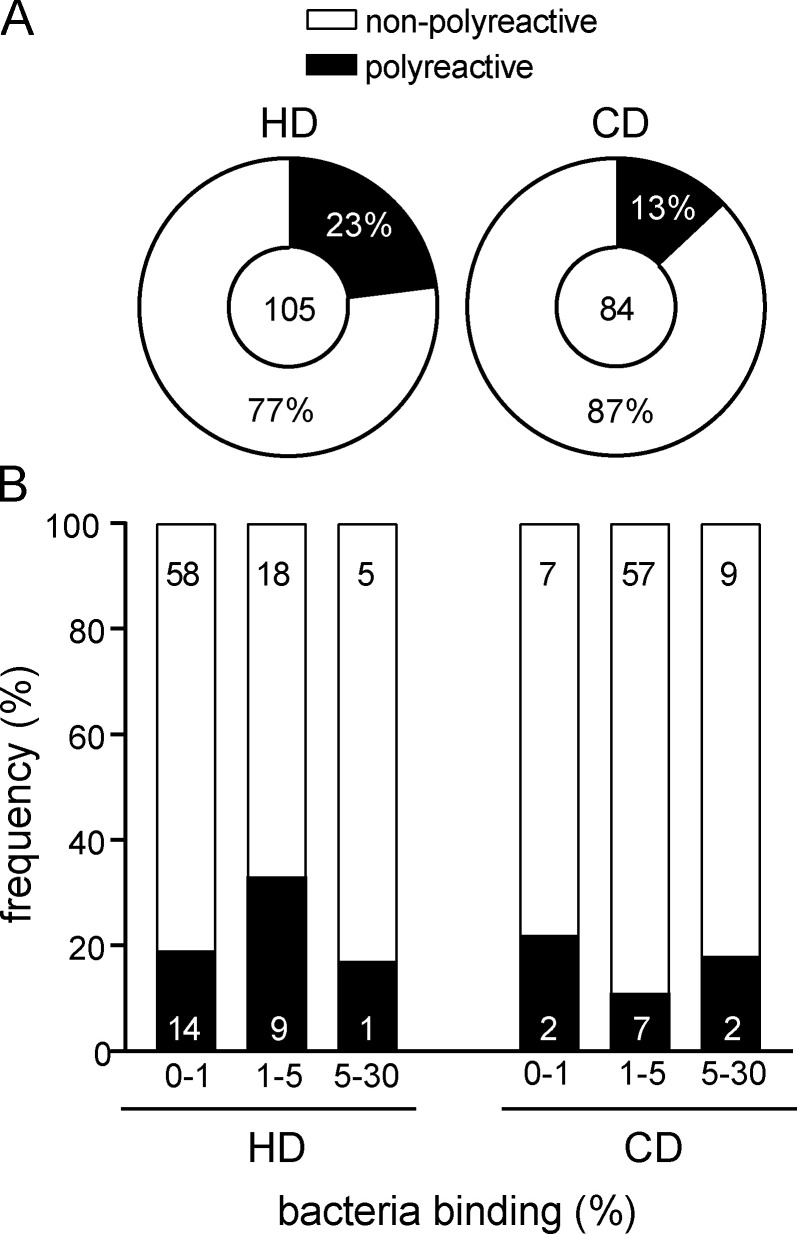
**High microbiota reactivity does not correlate with polyreactivity. (A)** IgA mAbs were tested in at least two replicate experiments for polyreactivity and classified as either polyreactive (black) or nonpolyreactive (white). **(B)** Bar charts show the relative distribution of polyreactive and nonpolyreactive HD-derived and CD patient–derived IgA antibodies among antibodies with no/low, intermediate, and high microbiota reactivity. Correlation between polyreactivity and microbiota reactivity was determined by linear regression (HD IgA, P = 0.97; CD IgA, P = 0.41).

To examine whether polyreactivity was an important determinant of cross-species reactivity of human intestinal mAbs, we screened the HD and CD collections of IgA- and IgG-derived antibodies for binding to a panel of seven unrelated antigens commonly used to test for polyreactivity. Antibodies that showed binding to more than two antigens were classified as polyreactive. Based on this criterion, 23% of IgA antibodies from HDs and 13% from CD patients were polyreactive (Fig. 5 A and Fig. S3 C; Benckert et al., 2011). Among IgG-derived antibodies, we found similar frequencies of polyreactive antibodies (23% for HD mAbs and 18% for CD; Fig. S3 D). Notably, polyreactive IgA and IgG antibodies were observed at similar frequencies among antibodies with no/low, intermediate, and high microbiota reactivity. In addition, polyreactive antibodies were not overrepresented among antibodies with high binding capacity for microbiota (Fig. 5 B and Fig. S3 D). Hence, correlation analysis did not reveal any positive relationship between polyreactivity and percentage of antibody-bound bacteria (HD IgA, P = 0.97; CD IgA, P = 0.41). Because we did not find an overrepresentation of polyreactive antibodies among high-microbiota-binding antibodies, we further investigated the possibility that the high capacity for microbiota binding of human intestinal IgA might rely on accumulated somatic mutations.

**Figure 6.**
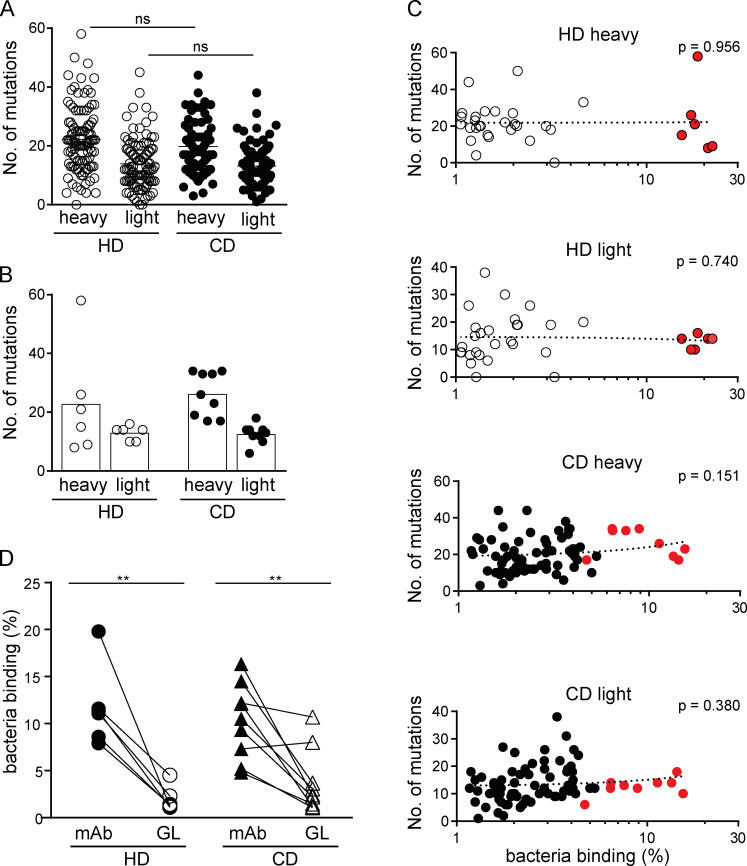
**Somatic mutations confer high microbiota**-**binding capacity.** **(A and B) **Numbers of somatic mutations in the heavy and light chain V genes displayed for all IgA antibodies obtained from HDs and CD patients (A) and for high-microbiota-reactive IgA antibodies (B). **(C)** Microbiota-binding capacity, displayed as percentage of bound microbiota determined as mean of two or more replicate experiments (for antibodies showing microbiota binding ≥1%), was correlated to the number of somatic mutations in heavy and light chain V genes for individual IgA antibodies. Mean microbiota-binding capacity of selected high-microbiota-reactive mAbs are shown in red. P values were obtained by linear regression analysis. **(D)** Pairwise comparison of microbiota-binding capacity of mAbs and their respective, predicted GL variant. Symbols represent the mean value of two independent experiments. Significance was tested by paired Mann–Whitney *U* test, **, P < 0.01; ns, not significant.

IgA- and IgG-derived antibodies from both HDs and CD patients showed the typical pattern of highly mutated intestinal antibodies in both heavy and light chain V gene sequences (Barone et al., 2011; Benckert et al., 2011; Berkowska et al., 2015; Lindner et al., 2015; Fig. 6 A and Fig. S3 E). No major difference was detected when comparing the number of mutations in the nonselected collection of antibodies to the set of antibodies with high microbiota-binding capacity (Fig. 6, A and B). The overall number of mutations in heavy or light chains of IgA-derived and IgG antibodies did not correlate with their capacity to bind gut bacteria (Fig. 6 C), with the exception of a positive correlation in HD-derived IgG light chains (Fig. S3 F).

**Figure S4.**
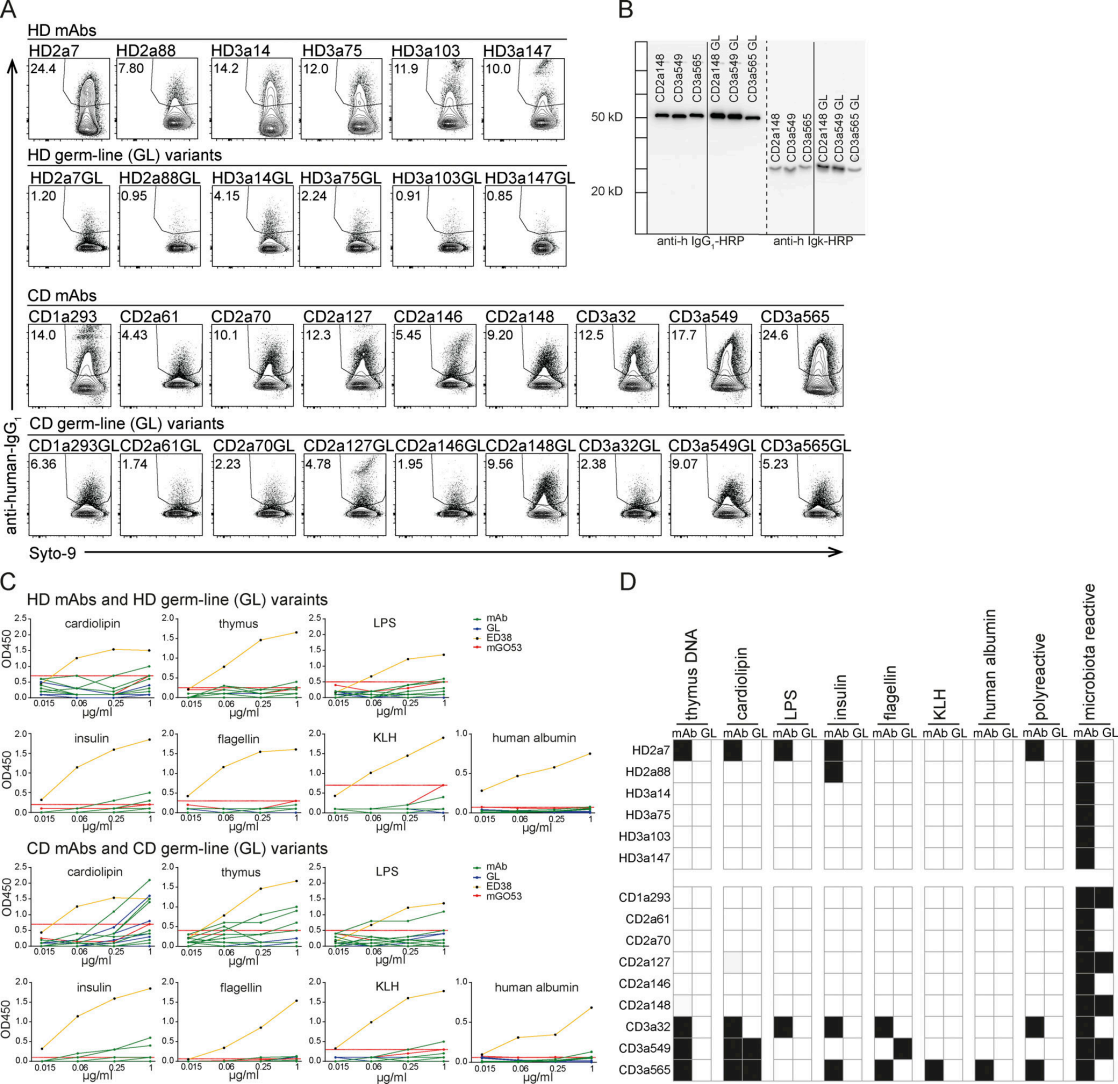
**Somatic mutations confer high microbiota**-**binding capacity. (A)** Representative FACS plots showing staining of bacteria (gated on forward/side scatter profile and Syto9-positive events) with IgA mAbs carrying somatic mutations compared with staining of bacteria with antibodies in their predicted GL configuration. Numbers indicate percentage of gated events. **(B)** Representative Western blot analysis to illustrate correct mass of heavy and light chains of antibodies before and after GL reversion. **(C)** Polyreactivity ELISA (OD_450_) showing the binding of selected HD and CD IgA mAbs and their GL variants to the standard set of seven unrelated antigens (insulin, cardiolipin, flagellin, KLH, LPS, albumin, and calf-thymus DNA). Antibodies were considered polyreactive when binding to more than two unrelated antigens above cutoff (dashed red line: OD_450_ of mGO53 at 1 µg/ml minus background). **(D)** Heat map representation of polyreactivity ELISA. Polyreactive mAbs or polyreactive GL variants are indicated by black squares. Data are representative of two independent experiments.

To directly address the contribution of somatic mutations to microbiota-binding capacity as well as specificity, we generated the predicted GL variants of IgA antibodies with high microbiota-binding capacity (Fig. S4, A and B). For all reverted HD-derived antibodies and for the majority of antibodies (seven of nine) derived from CD patients, the GL variant showed a significant loss of microbiota binding compared with the mutated variant (Fig. 6 D and Fig. S4 A). Our data therefore strongly suggest that in the human gut, somatic mutations significantly contribute to microbiota-binding capacity and cross-species reactivity of IgA. Importantly, GL reversion of antibodies did not render antibodies polyreactive. In fact, the polyreactive antibodies HD2a7, CD3a32 and CD3a565 lost polyreactivity after GL reversion (Fig. S4, C and D), indicating that these antibodies might have become polyreactive during somatic hyper mutation and cross-species reactive mAbs did not derive from originally polyreactive antibodies.

**Figure 7.**
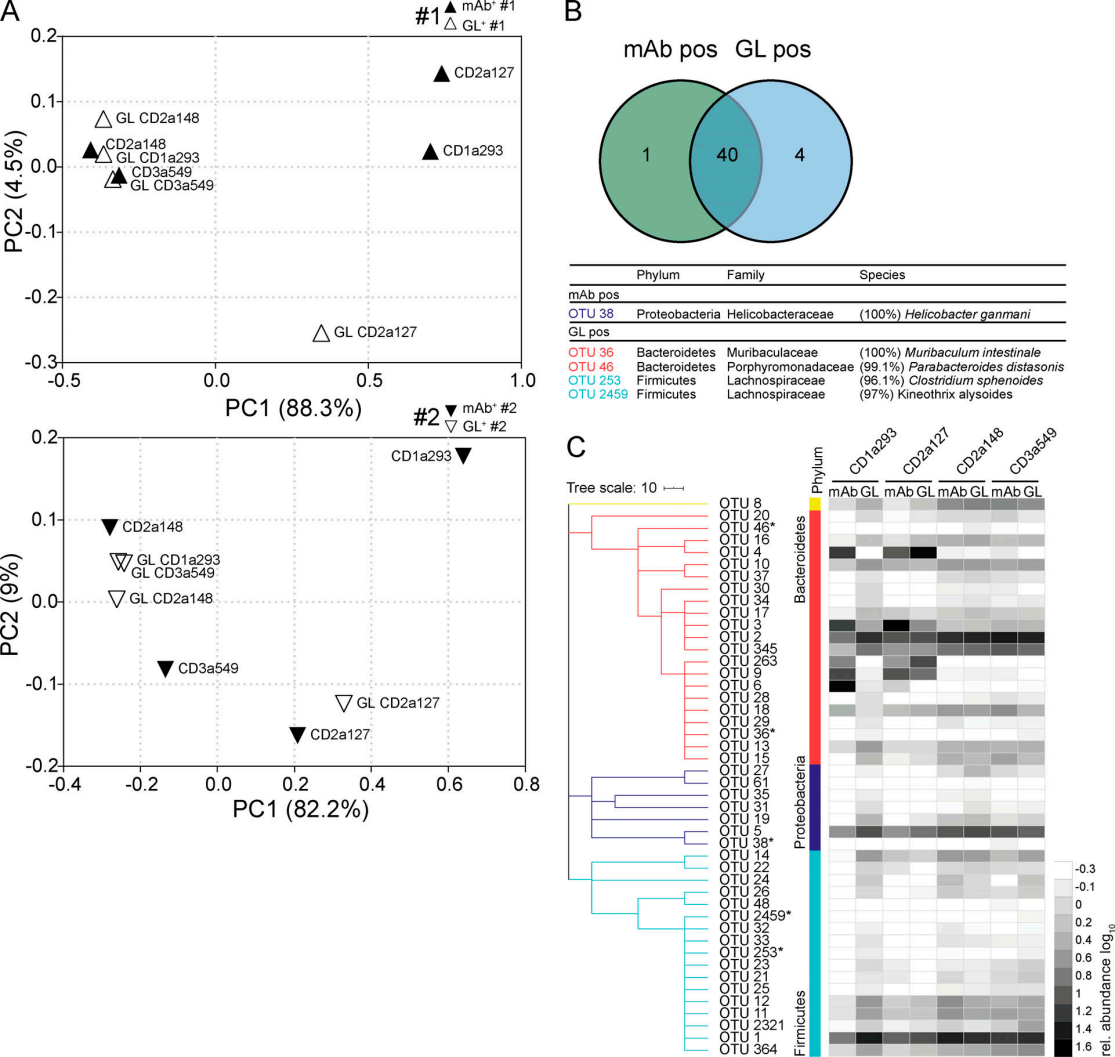
**Somatic mutations contribute to the gut microbiota specificity of human IgA.** Fecal bacteria bound by mAbs carrying somatic mutations and their corresponding GL variants were obtained by cell sorting and analyzed by 16S rRNA gene amplicon sequencing in two independent sets of experiments, #1 and #2. **(A)** β-Diversity PCoA plots based on generalized Unifrac distances depict comparison between sorted positive samples of mutated antibodies (filled symbols) and corresponding GL variants (open symbols). **(B)** Venn diagram depicts shared and selectively enriched OTUs in mAb-positive and GL-positive samples. Selectively enriched OTUs are marked by asterisks, EZbiocloud identified, and listed (Yoon et al., 2017). **(C)** Dendrogram clustering of OTUs organized (phylum annotations displayed in colors) based on the RDP taxonomic classification (Wang et al., 2007). Only OTUs occurring at a relative abundance ≥0.5% in any positive fraction are displayed. Heat map shows log_10_-transformed relative abundances of OTUs.

However, four CD-derived GL-variant mAbs retained sufficient microbiota-binding capacity to allow for sort-based purification of bound microbes. Two of these antibodies maintained or even showed increased binding capacity to bacteria (CD2a148 and CD3a549), and two mAbs showed reduced overall binding but still recognized a sortable bacterial population (CD1a293 and CD2a127) in their GL configuration (Fig. 6 D and Fig. S4 A). Mutated and GL antibodies largely bound the same bacterial species (Fig. 7 B), although often with differing efficiencies (Fig. 7 C). β-Diversity analysis of populations bound by the mutated antibodies and their corresponding GL variants showed close clustering for CD2a148 and CD3a549 and similar relative abundance of enriched OTUs (Fig. 7 A). There were notable differences in the relative binding capacities of mutated mAbs compared with their GL variants to multiple individual OTUs (Fig. 7 C). Moreover, for two of the mAbs, CD1a293 and CD2a127, the populations of bacteria bound by the mutated version and GL variant were substantially different (Fig. 7 C). For these two antibodies, some of the OTUs enriched by the mutated antibodies were less abundant in the population of bacteria bound by the GL variants, indicating that even though GL reversion retained some microbiota-binding capacity, mutations in these two antibodies contribute to their microbiota specificity and/or binding strength.

Collectively, these data show that fecal microbiota binding was unrelated to polyreactivity, whereas in most cases, GL reversion impaired microbiota-binding capacity or altered antibody specificity for the microbiota. Therefore, our data demonstrate that a system of affinity-matured IgA is one central feature of SIgA–microbiota interactions in the healthy and inflamed gut of adult humans.

## Discussion

Here we show that IgA and IgG antibodies derived from intestinal plasmablasts/plasma cells in adult humans can bind to a large proportion of fecal microbes. Such high microbiota-binding capacity was observed for different microbiota configurations, including murine samples as well as human samples obtained from either HDs or patients with IBD. High microbiota-binding capacity was associated with binding to a broad range of phylogenetically diverse bacteria. We refer to this mode of binding as cross-species reactivity (Pabst and Slack, 2020) and speculate that Ig binding to bacteria may constitute an important mechanism to regulate the gut microbiota and the integrity of the intestinal mucosa. Cross-species reactivity does not make any assumption about the mode of binding but points to interesting functional consequences for intestinal antibody function in the complex gut microbiota environment.

In the setting of a highly complex and dynamically changing ecosystem such as the gut microbiota, it is difficult to picture how antibodies selectively binding to single bacteria can be produced at functionally meaningful levels. How does the host generate sufficient amounts of secretory antibodies to affect the microbiota? A possible explanation is the concept of enchained growth, which refers to antibody-mediated cross-linking and agglutination of dividing bacteria (Moor et al., 2017). For instance, *Salmonella*-specific antibodies primarily cross-link dividing bacteria to enable agglutination of the pathogen in a situation in which few targeted bacteria are diluted within a complex and numerous endogenous microbiota (Moor et al., 2017). Cross-species–reactive antibodies might add to this concept by contributing to the agglutination of planktonic members of the microbiota.

Cross-species reactivity appears to be a feature of both mouse (Bunker et al., 2017) and human intestinal antibodies. However, although cross-species reactivity was proposed to be mainly achieved by “innate-like” polyreactive antibodies in mice (Bunker et al., 2017), we demonstrate here that, in adult humans, IgA cross-species reactivity typically depends on the accumulation of somatic mutations. It is possible that these two mechanisms of cross-species antibody reactivity may simply reflect a fundamental difference between the two hosts. However, we speculate that these seemingly contradictory findings may in fact reveal distinct facets of the intestinal immune response, which may act in concert to protect against pathogens and create microbiota-reactive antibodies throughout life in both mice and humans. At the outset, the newborn receives maternal antibodies that help shape the microbiota (Pabst et al., 2016), and provide protection while the immune system initially develops. Subsequently, in young individuals with relatively few germinal centers and little affinity maturation, polyreactive antibodies may be the dominant mechanism of microbiota binding. Indeed, such antibodies are prevalent in the gut of young mice (Bunker et al., 2017) and are present at low frequency in our collection of human antibodies. However, during aging, these may become supplanted by affinity-matured antibodies that accumulate somatic mutations, thereby enabling or even enhancing cross-species reactivity. Such antibodies are the distinguishing type of highly microbiota-reactive antibodies described here and are consistent with the well-documented finding of highly mutated plasma cell repertoires in the human gut exposed to a complex and dynamic environment and microbiota (Barone et al., 2011; Benckert et al., 2011; Lindner et al., 2015).

Intriguingly, cross-species reactivity does not extend to all members of the microbiota: some bacteria detected by sequencing did not show relevant binding by any of the tested antibodies, whereas others were commonly targeted by different mAbs or specifically enriched by single antibodies. Thus, intestinal antibodies display unique and specific binding profiles for distinct members of the gut microbiota. But what determines which bacterial taxa are targeted?

Polyreactive antibodies, per definition, bind unrelated molecules. Thus, binding of polyreactive antibodies to different bacterial species might function via unrelated antigens present on the different bacteria. The enrichment of defined bacterial taxa by intestinal antibodies as described in mice (Bunker et al., 2017) and here in humans suggests at least some level of antibody specificity. In contrast, hypermutated antibodies abundant in the human gut are likely to be the result of ongoing selection for high-affinity binding. Cross-species reactivity of high-affinity antibodies might rely on epitopes shared between different bacterial species such as conserved glycan structures or highly conserved peptides (Rollenske et al., 2018; Bunker et al., 2019; Diard et al., 2019 *Preprint*; Sterlin et al., 2020). The identification of epitopes targeted by IgA across multiple members of the microbiota is the crucial next step to further understand the phenomenon of cross-species reactivity and to dissect pathways of how cross-species reactivity is achieved.

Notably, bacteria commonly targeted by antibodies isolated from HDs were also bound by high-microbiota-reactive antibodies from CD patients. Therefore, selective broad binding to distinct OTUs seems to be a hallmark of intestinal IgA in both healthy and inflamed intestine. Thus, in both situations, the profile of IgA-targeted bacteria comprised commonly targeted OTUs in addition to specific members of the microbiota. These observations indicate that major changes in gut microbiota composition as observed in IBD patients do not necessarily dominate the microbiota specificity of IgA and are consistent with our previous observation that alterations of the microbiota do not rapidly result in major changes of the plasma cell repertoire (Lindner et al., 2015).

The presence of cross-species–reactive antibodies under healthy and inflamed conditions might hint at the functional importance of such antibodies. Cross-species reactivity may contribute to efficient agglutination of opportunistic pathogens, possibly including the endogenous microbiota as scaffolding. Yet proinflammatory functions can also be envisioned, and cross-species reactivity might further exacerbate the inflammatory response to members of the microbiota. In particular, in situations of an impaired intestinal barrier such as in IBD patients, cells in the lamina propria will encounter IgA-coated intestinal bacteria. IgA coating of the bacteria has the potential to modulate the ensuing immune response. Depending on the precise context, interaction with the IgA Fc receptor FcαRI can initiate proinflammatory or inhibitory responses (Bakema and van Egmond, 2011).

An important step in understanding the generation of microbiota-targeting Ig responses and cross-species reactivity might come from a deeper understanding of how somatic hypermutation shapes these antibodies. Indeed, our data suggest that accumulating somatic mutations fundamentally contribute to antibody cross-species reactivity. In adult humans, the continuous exposure to varied but structurally similar bacterial antigens may favor the proliferation and differentiation of B cells with cross-species specificity. In this context, an important step forward may come from a deeper understanding of which bacterial species contribute to the initial activation of the B cell response and subsequent affinity maturation. Indeed, when testing antibodies selected for high microbiota reactivity to murine fecal samples, these antibodies also showed high microbiota reactivity to human fecal material from HDs and patients with IBD. This suggests that either closely related bacterial strains are present in all samples or that intestinal antibodies are indeed directed against structurally similar bacterial antigens shared among different members of the microbiota, independent of the host.

Collectively, our data highlight a new facet of IgA: high microbiota and cross-bacterial species reactivity of human intestinal IgA rely on somatic mutations. We propose that cross-species-specific antibodies provide a mechanism to efficiently interact with multiple microbial members and represent one of the major mechanisms by which the intestinal immune system interacts with the intestinal microbiome.

## Materials and methods

### Mice

Rag2-deficient mice and C57BL/6J WT mice were bred and reared at RWTH Aachen University under specific pathogen–free (SPF) conditions. Germ-free C57BL/6J mice were bred and reared at the Laboratory for Animal Sciences at Hannover Medical School. Animal work was performed in compliance with ethics regulations of German Law for the Protection of Animal Welfare (Tierschutzgesetz) and approved by North Rhein-Westphalia State Agency for Nature, Environment and Consumer Protection (Landesamt fur Natur, Umwelt und Verbraucherschutz Nordrhein-Westfalen).

### Human fecal material

The human biomaterials were provided by the centralized biomaterial bank of the Medical Faculty of RWTH Aachen University (RWTH cBMB) and were used in accordance with the regulations of the RWTH cBMB and the Ethics Vote 206/09 of the Ethics Committee of the Medical Faculty of RWTH Aachen University.

### Production of mAbs

Generation of mAbs from three HDs undergoing right-sided hemicolectomy has been described before (Benckert et al., 2011). None of the donors had a history of intestinal inflammation, and the samples showed no signs of inflammation as determined by macroscopic evaluation and histopathologic examination of the adjacent mucosa (Benckert et al., 2011). A second collection of antibodies was generated from surgical samples of three CD patients undergoing ileocoecal resection (CD1, male, 25 yr old; CD2, male, 47 yr; CD3, male, 22 yr) and were obtained after signed informed consent in accordance with protocols reviewed and approved by the Charité University Hospital institutional review board (EA1/257/12).

### Single B cell sorting

Intestinal plasmablasts/plasma cells were isolated as previously described (Benckert et al., 2011). In brief, lamina mucosa and propria were dissected from lamina muscularis mucosae by blunt preparation, and 2–3-mm tissue pieces were digested using 0.1% DNase and 0.1% collagenase followed by discontinuous Ficoll density gradient centrifugation (GE Healthcare). Purified lamina propria lymphocytes were stained on ice with fluorochrome-coupled anti-human CD38 FITC, anti-human CD27 PE, anti-human CD19 PECy7, anti-human IgG APC (all from BD Bioscience) or anti-human IgA APC (Jackson Laboratory). Single CD38^+^CD27^+^IgA^+^ or CD38^+^CD27^+^IgG^+^ plasmablasts/plasma cells were sorted into 96-well PCR plates using a FACSVantage cell sorter with Diva configuration (BD Bioscience), snap frozen on dry ice, and stored at −80°C until further processing.

### PCR amplification and expression vector cloning

Single-cell cDNA synthesis and nested PCR amplification of *IGG* or *IGA* and *IGK* or *IGL* genes was performed as described (Benckert et al., 2011). All PCR products were sequenced before and after cloning into previously described eukaryotic expression vectors (Tiller et al., 2008). In brief, PCR products of IgH V(D)J and *IGK* Igk and *IGL* Igl VJ variable regions were cloned into separate expression vectors encoding for the constant regions of the human IgG1, *IGK* and *IGL* light chain to allow for expression of all antibodies as fully human Fc-IgG1 antibodies. Ig gene sequence analysis, including Ig gene usage, clonal relationships, IgG subclass, somatic mutations, IgH CDR3 length, and positive charged amino acids, was performed using IgBlast (http://www.ncbi.nlm.nih.gov/igblast/).

### Expression and purification of recombinant antibodies

239T HEK cells were transiently transfected with equal amounts of both corresponding IgH and IgL chain plasmids using a calcium phosphate kit (Invitrogen; K2780-01). Transfected HEK cells were cultured in Pro 293a Media (Lonza) supplemented with 1 mM sodium pyruvate, 2 mM l-glutamine, and 100 U/ml penicillin G–streptomycin (all from Gibco). Supernatants were collected 60 and 72 h after transfection and filtered through a sterile filter (0.20 µm) and stored at 4°C. Igs were purified from supernatants using Protein A–based affinity chromatography (Äkta Start; GE Healthcare Life Sciences). Protein A HiTrap columns (GE Healthcare Life Sciences) were loaded with Ig-containing supernatant and washed with 20 mM sodium-phosphate buffer, and antibodies were eluted using 0.1 M citric acid (pH 3.0), pH neutralized with 1 M Tris-HCl (pH 9.0). Collected supernatants were concentrated and washed with PBS, and antibodies were collected in PBS. Concentrations of purified antibodies were determined by ELISA.

### Deglycosylation of antibodies

To remove human IgG Fc N-glycan moieties by deglycosylation, GLycINATOR (EndoS2) an endoglycosidase for hydroxylation of β1,4 linkage of core GlcNAc residues, was used according to the manufacturer’s instructions (GlycINATOR R; Genovis). Deglycosylation was performed with 1 unit of GlycINATOR/1 µg IgG and incubated for 30 min at 37°C. Deglycosylation was confirmed by mass spectrometry and Western blot. Samples were reduced with Tris(2-carboxyethyl)phosphine before electrospray ionization mass spectrometry analyses, desalted using a C4 ZipTip (Millipore), and analyzed in MeOH:2-PrOH:0.2% FA (30:20:50). The solutions were infused through a fused silica capillary (internal diameter, 75 µm) at a flow rate of 1 µl/min and sprayed through a PicoTip (internal diameter 30 µm; both from New Objective). Nano-electrospray ionization mass spectrometry analyses of the samples was performed on a Synapt G2_Si mass spectrometer, and the data were recorded with MassLynx 4.2 software 2/2 (both from Waters). Mass spectra were acquired in the positive-ion mode by scanning an m/z range from 100 to 5,000 daltons with a scan duration of 1 s and an interscan delay of 0.1 s. The spray voltage was set to 3 kV, source temperature 80°C, and cone voltage 50 V. The recorded m/z data were then deconvoluted into mass spectra by applying the maximum-entropy algorithm MaxEnt1 (MaxLynx) with a resolution of the output mass 0.5 daltons/channel and Uniform Gaussian Damage Model at the half height of 0.7 daltons.

Deglycosylation efficiency of human IgG1 was independently analyzed by Western blot. Heat-denatured samples were separated on a 12% acrylamide gel (Bio-Rad) and transferred onto a nitrocellulose membrane (0.45 µm, Bio-Rad; 1620115). For detection of IgG1 heavy chains, membranes were blocked with 5% milk powder in PBS/0.1% Tween-20 overnight at 4°C and incubated with HRP-conjugated anti-IgG1-HRP antibody (Jackson ImmunoResearch; 109-035-098) for 1 h. To detect glycans, membranes were blocked with Hepes 2% Tween-20 buffer, washed twice with Hepes, and incubated with concanavalin-A–HRP for 16 h at room temperature (0.2 µg/ml in Hepes, 0.05% Tween-20, and 1 mM CaCl_2_, MgCl_2_, MnCl_2_, and concanavalin A [Sigma-Aldrich; L6397]). HRP activity was detected with ECL substrate (Bio-Rad).

### ELISA

The concentration of recombinant antibodies was determined by ELISA. ELISA 96 plates (NUNC Maxi Sorp) were coated with 1.25 ng/ml of anti-human IgG Fc (AffiniPure goat anti-human IgG, Fcγ-specific; Jackson ImmunoResearch; 109-005-098) in PBS. Plates were washed with PBS/0.05% Tween-20 and blocked with PBS/2% BSA for 1 h at 37°C. Human serum IgG_1_ standard (Sigma-Aldrich) and recombinant antibodies diluted in PBS/0.05% Tween-20 at twofold dilution were incubated for 1.5 h at 37°C and detected with HRP-conjugated (Peroxidase AffiniPure goat anti-human IgG, Fcy-specific; Jackson ImmunoResearch, 109–035-098) diluted in PBS/0.05% Tween-20 at a concentration of 0.8 µg/ml (Jackson ImmunoResearch Laboratories) according to the manufacturer’s instructions. ODs were measured at 450 nm, and antibody concentrations were determined using the SpectraMax microplate reader and software (Molecular Devices) and Excel 2010 (Microsoft).

### Generation of GL variants

Sequences were analyzed by IgBLAST comparison with GenBank, and GL segments with the highest probability were determined. The reverted sequences were generated as gBlock double-stranded DNA fragments from IDT (Integrated Technologies) with included flanking primer and restriction sites. GL (gBlock) fragments (100 ng/µl) and original vector plasmids containing IgH and IgL sequences were digested with respective restriction enzymes (all from NEB). Vector backbone was purified by gel electrophoresis in a 1.8% agarose gel, and gBlocks were purified by PCR purification Kit (Qiagen). Ligation was performed with T4 DNA ligase (NEB), and products were transformed into competent 5-α *E. coli* cells (NEB). For each antibody, several clones were analyzed, and the GL and original insert sequences were confirmed with IgBLAST comparison with GenBank and Gentle free software (GENtle). The molecular weight of GL variants was validated by Western blot analysis for heavy and light chains according to standard procedures.

### Bacterial flow cytometry and bacterial sort purification by FACS

Fecal pellets from SPF Rag2-deficient and germ-free WT mice were collected and human fecal material (from five HDs and five ulcerative colitis patients) was obtained and stored at −80°C. Murine fecal pellets or aliquots from human feces were homogenized in 1.5-ml tubes in 1 ml of filtered (0.2 µm) HBSS. Homogenized samples were centrifuged at 500 rpm for 1 min, and the process was repeated until the supernatant was clear of debris. Clear supernatant was centrifuged at 8,000 rpm (VWR Microstar 17R) for 8 min, and the supernatant was discarded. The bacterial pellet was resuspended in 100 µl of filtered HBSS/2% normal goat serum (Sigma-Aldrich) and passed through a sterile cell strainer (40 µm). The OD_600_ was determined in a 1:100 dilution, and suspensions were adjusted to OD_600_ of 0.13 ± 0.01 or 0.2 ± 0.01 for human fecal suspensions (5 × 10^7^/ml bacteria). Antibody concentrations were adjusted to 1 µg/ml in HBSS/2% normal goat serum. 2 µl of murine bacterial suspensions were stained with 100 µl of diluted antibody, incubated on ice for 20 min, and washed in 1 ml HBSS/2% goat serum at 13,000 rpm for 8 min at 4°C. The supernatant was discarded, and pellets were resuspended in 100 µl anti-human IgG_1_ AF647 (1:200; 0.5 mg/ml, Alexa Fluor 647–conjugated AffiniPure Goat, anti-human IgG [H + L], Jackson ImmunoResearch) and incubated for 20 min on ice. 50 µl of Syto9 dye (Syto 9 green fluorescent nucleic acid stain, 5 mM in DMSO; Thermo Fisher Scientific) in HBSS was added and incubated for 10 min on ice. For bacterial suspensions isolated from human fecal material, 2 µl of bacterial suspensions were stained with 5 µg/ml AF647-directly conjugated mAbs for 20 min on ice followed by Syto9 staining and washing as described for murine samples. A minimum of 100,000 Syto9-positive events were acquired for each sample. For FACS purification, samples were prepared as described above in replicates, pooled, and sorted on a FacsAria II (BD). Sorted samples were centrifuged at 13,000 rpm for 8 min at 4°C, and pellets were stored at −80°C.

### 16S rRNA gene amplicon sequencing and analysis

Bacterial DNA from human fecal samples was extracted via glass bead preparation and further isolated using the QIAamp DNA Stool Kit (Qiagen) according to the manufacturer’s protocol. Metagenomic DNA of sort-purified murine bacterial samples of mAb-positive and mAb-negative fractions and input material was extracted using the Qiagen DNA isolation kit according to the manufacturer’s instructions. OTU profiles obtained for fecal material and isolated bacteria were highly comparable (not depicted). The V3/V4 regions of 16S rRNA genes were amplified (35 cycles) using primers 515F and 816R. PCR amplicons were purified by agarose gel electrophoresis (1.5%) followed by purification using a QIAquick Gel Extraction Kit (Qiagen). Empty samples were included in the overall sample processing (FACS buffer collected during cell sorting) and analyzed along with H_2_O and buffer controls used during library preparation to identify potential artifact sequences. DNA was quantified using the Quant-iT PicoGreen dsDNA Kit assay (Invitrogen), and PCR products were stored at −20°C until sequencing. The amplicon libraries were sequenced with single barcodes in paired-end mode (2 × 300 nt) using MiSeq sequencer (Illumina Miseq; Uniklinik/RWTH Aachen). Raw reads were processed using the IMNGS platform based on the UPARSE approach (Edgar, 2013). OTUs were clustered at a sequence identity of 97%, and only OTUs occurring at a relative abundance >0.25% in at least one sample were used for further analyses. Taxonomic assignment was based on the Ribosomal Database Project (RDP) classifier v2.11 (Wang et al., 2007) with a confidence cutoff of 80%, and dendrogram clustering of OTUs (phylum to family) was based on genomic sequence similarity obtained by the RDP taxonomic classification (Wang et al., 2007). The lineage and taxonomic identity (closest species with a valid name and corresponding 16S rRNA gene sequence identity) of relevant OTUs were obtained using EZbiocloud (Yoon et al., 2017). Diversity and sample composition analyses were performed using Rhea v2.0 in R (Lagkouvardos et al., 2017). All 16S rRNA data are freely available under BioProject accession no. PRJNA622437.

### Polyreactivity ELISA

Polyreactivity assays were performed as described previously (Benckert et al., 2011). In brief, ELISA plates were coated with 50 µl of the respective antigens (10 µg/ml calf thymus DNA, 5 µg/ml human recombinant insulin, 10 µg/ml LPS, 5 µg/ml flagellin, 10 µg/ml cardiolipin, 10 µg/ml human albumin, and 10 µg/ml KLH) in PBS, overnight at 4°C. ELISA plates were washed three times with PBS and incubated with 50 µl of antibody at 1, 0.25, 0.06, and 0.015 µg/ml in PBS for 1 h. Recombinant human mAbs mGO53 (nonpolyreactive; Wardemann et al., 2003) and ED38 (high polyreactive; Meffre et al., 2004) were used for comparison on each plate. ELISA plates were developed with HRP-labeled goat anti-human IgG Fcy specific antibody (Jackson ImmunoResearch; 109-035-098) at a concentration of 0.8 µg/ml with 2 mM EDTA and 0.05% Tween-20 and HRP chromogenic substrate (ABTS; Pierce). OD_450_ was measured using a microplate reader (Molecular Devices) and Soft-Max software (Molecular Devices). The cutoff value for each plate was determined based on the highest OD_450_ value at a concentration of 1 µg/ml of the nonpolyreactive control mAb (mGO53). Antibodies showing binding to at least two different antigens were considered polyreactive.

### Statistical analysis

Statistical analysis for FACS-generated data was performed with GraphPad Prism 7. P values ≤0.05 were considered significant. Statistics for 16S rRNA gene amplicon analyses are described in detail in Rhea (https://lagkouvardos.github.io/Rhea/; Lagkouvardos et al., 2017).

### Online supplemental material

Fig. S1 shows representative FACS plots of bacterial staining with selected high-microbiota-reactive mAbs from HDs and CD patients and mAb staining quantification of bacterial material obtained from unrelated feces or aliquots of pooled fecal material. Fig. S2 shows log_10_-transformed relative abundance of all OTUs in HD and CD mAb-positive and -negative samples and input material, with OTUs phylogenetically organized as a dendrogram. Fig. S3 shows representative FACS plots of binding capacity of mAbs or their deglycosylated counterparts and quantification of independent experiments; summary of polyreactivity and microbiota reactivity for all tested IgA and IgG mAbs derived from HDs and CD patients; overall distribution of polyreactive IgG mAbs among the HD and CD antibody collections tested; relative distribution of polyreactive and nonpolyreactive IgG mAbs among no/low, intermediate, or high microbiota-reactive mAbs; absolute number of mutations in IgG H and L V genes; and correlation of number of mutations in Ig genes to microbiota-binding capacity for HD- and CD-derived IgG mAbs. Fig. S4 shows representative FACS plots of microbiota-binding capacity of selected high-microbiota-reactive HD- and CD-derived IgA mAbs before and after GL reversion; representative Western blot analysis to validate the molecular weight of heavy and light chains; polyreactivity ELISA representations for selected high-microbiota-reactive HD- and CD-derived IgA mAbs before and after GL reversion tested against seven unrelated antigens; and a summary heat map of polyreactivity and microbiota reactivity of selected high-microbiota-reactive HD- and CD-derived IgA mAbs before and after GL reversion. Table S1 contains Ig sequence analysis of CD-derived IgA^+^ and IgG^+^ intestinal plasmablasts/plasma cells and microbiota reactivity of tested mAbs. Table S2 lists taxonomic classification of all identified OTUs in HD and CD mAb–positive and –negative samples and input material.

## Supplementary Material

Table S1 shows Ig gene sequence analysis of CD patient–derived IgA^+^ and IgG^+^ intestinal plasmablasts/plasma cells.

Table S2 shows the taxonomic classification of detected OTUs based on 16S rRNA gene amplicon sequence identity.
